# Bioreactor Expansion Affects Microbial Succession of Mixotrophic Acidophiles and Bioremediation of Cadmium-Contaminated Soils

**DOI:** 10.3390/toxics12050362

**Published:** 2024-05-13

**Authors:** Xiaodong Hao, Ping Zhu, Xueduan Liu, Luhua Jiang, Huidan Jiang, Hongwei Liu, Zhiqun Chen

**Affiliations:** 1 Shandong Provincial Key Laboratory of Water and Soil Conservation and Environmental Protection, College of Resources and Environment, Linyi University, Linyi 276000, China; haoxiaodong@lyu.edu.cn (X.H.); zhuping199508@163.com (P.Z.); 2 School of Minerals Processing and Bioengineering, Central South University, Changsha 410083, China; xueduanliucsu@163.com (X.L.); jiangluhua@csu.edu.cn (L.J.); 3 Biotechnology Research Institute, Hunan Academy of Agricultural Sciences, Changsha 410125, China; huidanjiangcsu@163.com; 4 College of Life Science, Linyi University, Linyi 276000, China

**Keywords:** scale-up cultivation, mixotrophic acidophiles, microbial community dynamics, Cd removal, indirect effect

## Abstract

Microbial scale-up cultivation is the first step to bioremediating cadmium (Cd)-contaminated soils at the industrial scale. However, the changes in the microbial community as the bioreactor volume expands and their associations with soil Cd removal remain unclear. Herein, a six-stage scale-up cultivation process of mixotrophic acidophiles was conducted, scaling from 0.1 L to 10 m^3^, to remediate Cd-contaminated soils. The findings showed that bioreactor expansion led to a delay in sulfur and glucose oxidations, resulting in a reduced decline in solution pH and cell density. There were minimal differences observed in bacterial alpha-diversity and community structure as the bioreactor volume increased, except for the 10 m^3^ scale. However, bioreactor expansion decreased fungal alpha-diversity, changed the community structure, and simplified fungal community compositions. At the family level, *Acidithiobacillaceae* and *Debaryomycetaceae* dominated the bacterial and fungal communities throughout the scale-up process, respectively. Correlation analysis indicated that the indirect effect of mixotrophic acidophiles played a significant role in soil Cd removal. Bacterial community shifts, driven by changes in bioreactor volume, decreased the pH value through sulfur oxidation, thereby indirectly enhancing Cd removal efficiency. This study will contribute to the potential industrial application of mixotrophic acidophiles in bioremediating Cd-contaminated soils.

## 1. Introduction

Soil cadmium (Cd) contamination has become a global environmental concern resulting from industrialization, urbanization, and unsustainable farming practices [1,2,3]. Even at low concentration, Cd is highly toxic and carcinogenic [4]. While Cd is resistant to degradation by organisms, it is easily accumulated in soils and crops, and subsequently enters the human food chain [5]. This poses a significant threat to environmental ecology and human health [6]. Therefore, it is imperative to remediate Cd-contaminated soils to mitigate the risks associated with Cd exposure and ensure food security.

The soil Cd mobilization strategy, compared to the Cd stabilization technique, focuses on minimizing Cd pollution by transforming Cd speciation into dissolved fractions and/or directly extracting Cd from soils [7]. Phytoextraction is an economic and ecological approach for removing Cd from soils [8]. However, the extensive application of phytoremediation is limited by factors such as a long soil restoration time, slow plant growth, and variable soil and weather conditions. Microbial extraction (bioleaching) is a promising approach for the efficient extraction of heavy metals from solid materials. It involves the direct biocorrosion of microbial metabolism or the indirect solubilization of microbial metabolites [9]. Bioleaching technology has been extensively studied due to its cost-effectiveness, eco-friendliness, and effective resource recovery. It has been successfully applied for in situ processing of copper sulfide ores at an industrial scale and for recovering valuable metals from various secondary sources at a pilot scale [10,11]. The employment of microbial extraction technology in large-scale remediation efforts for Cd-contaminated soils worldwide holds significant promise for enhancing the sustainability and efficiency of the soil treatment process. However, the application of bioleaching for Cd bio-extraction from heavy metal-contaminated soils is limited to laboratory-level studies, mainly due to factors such as low microbial functional diversity and slow dissolution kinetics [12]. Consequently, the selection of appropriate microbial functional flora and the provision of supporting hardware facility and cultivation condition are crucial for ensuring the feasibility and functionality of the scale-up cultivation of microbes.

The microbial composition is the most important factor influencing the extraction rate of heavy metals in bioleaching systems. Autotrophic bacteria and heterotrophic fungi have demonstrated their capacity to dissolve Cd complexes in soils through processes such as displacement of Cd ion by hydrion or the formation of chelate through the microbial production of inorganic/organic acids, leaching agents, biosurfactants, and other metabolites [13,14]. Cd extraction from soil matrices using pure species or monotrophic cultures typically involves the solubilization of Cd fractions in specific areas [15]. However, the bioleaching efficiency, when using mixed cultures, is higher for the same Cd fraction compared to that of a single population. Recently, mixotrophic acidophiles consisting of autotrophic and heterotrophic communities with supernally diverse phylogenetic and functional characteristics have been found to exhibit high Cd removal capability from soils [16]. In cooperative bioleaching systems involving the symbiotic co-culture of bacterial and fungal consortia, mixotrophic acidophiles exhibit significant potential in enhancing the extraction efficiency of soil Cd [17].

The successful industrial application of bioleaching relies on the scale-up cultivation of microorganisms. Bioreactor cultivation offers numerous advantages over traditional culture tanks, including better control of physicochemical properties in the solution and the provision of suitable growth conditions [18,19]. The microbial community structure in the scale-up cultivation of mixotrophic acidophiles is closely linked to key biochemical functions involved in the activation and extraction of Cd from soils [20]. As the bioreactor volume increases, microbial growth necessitates adaptation to larger spaces and environmental fluctuations, which can affect their resistance to environmental stress. Larger bioreactors require more time to achieve uniform distribution of nutrients and energy substrates, influencing microbial growth rates and distribution and thereby impacting the microbial community structure. Concurrently, with increased volume, dissolved oxygen concentrations may decline, impacting the growth of aerobic and oxygen-demanding microorganisms. The expansion of the bioreactor also affects the production of microbial metabolites, as larger spaces enable microorganisms to produce more products at different stages of metabolism, thereby influencing the microbial community’s functionality [21]. Presently, research on the effects of bioreactor expansion on changes in microbial community structure has primarily focused on the liquid fermentation process [22]. Limited work has been conducted to examine microbial community successions in the context of increasing bioreactor volume, especially regarding the complex mixotrophic community at different scale-up cultivation stages. The investigation of the broad applicability of the functional microbial population across diverse geographical locations and climatic environments is important for the implementation of microbial expansion technology in the soil remediation field.

To address this gap, the present study aims to investigate the following questions: (i) to measure the effects of bioreactor expansion on the microbial community successions of mixotrophic acidophiles; and (ii) to evaluate the effects of changes in solution properties and microbial structure on the soil Cd removal efficiency. Furthermore, the contribution of bioreactor parameters to soil Cd extraction, considering the direct and indirect actions of microbes and solution properties, also requires further analysis, which will aid in adjusting bioreactor parameters to maintain a stable microbial community structure and ensure efficient Cd bioleaching.

## 2. Materials and Methods

### 2.1. Initial Inoculum for the Scale-Up Cultivation of Mixotrophic Acidophiles

Leachate and Cd-contaminated soils were utilized to enrich the initial inoculum for the scale-up cultivation of mixotrophic acidophiles. The leachate sample was collected from a bioleaching heap of sulfide minerals in Dexing City, Jiangxi Province, China (29°04′ N, 117°71′ E). The Cd-contaminated soil was obtained from a polluted rice-growing region in Xiangtan County, Hunan Province, China (27°77′ N, 112°88′ E). The leachate (10 mL) and fresh soil (10 g) samples were combined and added to a 250 mL conical flask containing 100 mL of basal medium. The basal medium composition included 3 g/L (NH_4_)_2_SO_4_, 0.1 g/L KCl, 0.5 g/L K_2_HPO_4_, 0.5 g/L MgSO_4_·7H_2_O, 0.01 g/L Ca(NO_3_)_2_, 1 g/L elemental sulfur, 0.7 g/L glucose, and 0.3 g/L yeast extract. The solution’s pH value was adjusted to 3.5 using sulfuric acid, and then incubated in an oscillating incubator at 32 °C and 175 rpm. When the solution’s pH dropped below 2.5, the supernatant microbial fluid was transferred to a new basal medium (10%, vol/vol) and cultured under the same conditions. This process of uniform subculturing was repeated 10 times, and the microbial solution from the tenth culture was used as the initial inoculum for the scale-up cultivation of mixotrophic acidophiles.

### 2.2. Scale-Up Cultivation Process of Mixotrophic Acidophiles

**Figure 1:**
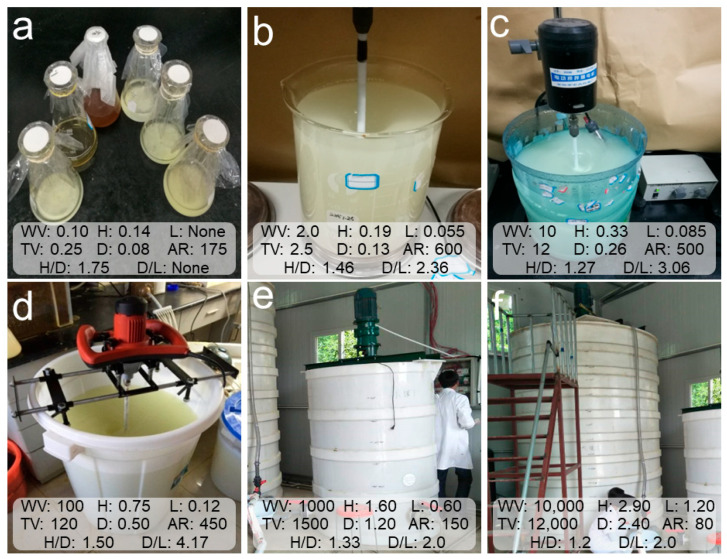
Bioreactor parameters used for the scale-up cultivation process of mixotrophic acidophiles. (**a**–**f**) Scale-up cultivation stages of 0.1 L, 2 L, 10 L, 100 L, 1 m^3^, and 10 m^3^, respectively. WV, work volume (L); TV, total volume (L); H, bioreactor height (m); D, bioreactor diameter (m); L, paddle length (m); AR, agitator agitation rate (rpm).

A six-stage scale-up cultivation system, including the lab-scale (0.1 L and 2 L), pilot-scale (10 L and 100 L), and commercial-scale (1 m^3^ and 10 m^3^) cultivation stages, was designed for the scale-up cultivation of mixotrophic acidophiles. These cultivation processes were conducted in vessels of varying sizes: a 250 mL glass conical flask; a 2.5 L glass beaker; and 12 L, 120 L, 1.5 m^3^ and 12 m^3^ cylindroid polyethylene reactors. The details of the bioreactor parameters are shown in Figure 1. The initial inoculum served as the seed liquid of the 0.1 L cultivation system. The scale-up process of mixotrophic acidophiles was continuous, where the microbial solution from a smaller cultivation stage acted as the seed liquid for the next stage once the solution pH dropped below 2.5 or the cell density exceeded 1 × 10^9^ cells/mL. The seed liquid dose was 10% (vol/vol). The cultivation media used in each stage were identical to the basal medium used for the initial inoculum, maintaining a constant temperature of 32 °C and an initial pH of 3.5. Each cultivation stage lasted approximately four to five days. Finally, the microbial solutions from each of the six scale-up cultivation stages were collected separately for microbial community analysis and soil Cd removal experiments.

### 2.3. Bioremediation of Cd-Contaminated Soils

The Cd-contaminated soils used in this study had been exposed to industrial sewage for approximately ten years. The soil type was clay. Top soil samples (0~20 cm) were collected using a T-sampler. Fresh soils were utilized to enrich the initial inoculum, as described in Section 2.1. The remaining soil samples were air-dried and passed through a 2 mm plastic sieve for the soil bioremediation experiment and the measurement of soil chemical properties (Table 1). The soil bioremediation experiment was conducted in 250 mL conical flasks. A soil sample (10 g) and microbial solution (100 mL) from the six scale-up cultivation stages were added to the conical flasks. To account for the potential impact of pH on soil Cd removal efficiency, a control treatment (CK) using the basal medium with a pH of 2.5 was established. The flasks were incubated in an oscillating incubator at a consistent temperature of 32 °C and a rotational speed of 175 rpm for 7 days. Each treatment was replicated three times. At the end of the bioremediation experiment, soil residue samples were obtained by centrifuging at 3000× *g* for 10 min. The supernatants were discarded, and the solid residues were air-dried. Total Cd and DTPA-Cd contents in the soil residues were measured to calculate the Cd removal efficiencies.

### 2.4. Chemical Analysis

The pH values of microbial solutions in each scale-up cultivation system were monitored daily using a pH meter (BPH-220, Bell Instrument, Dalian, China). The sulfate radical concentration and glucose concentration in the microbial solution were analyzed at the beginning and end of each scale-up cultivation performance. The sulfate radical concentration was measured using the barium sulfate precipitation method [23]. The glucose concentration was determined using the Agilent 1200 HPLC (Agilent Technologies Co. Ltd., USA). The increased sulfate radical concentration (ISC) was calculated as the difference between the sulfate radical concentrations after and before the cultivation performance, while the decreased glucose concentration (DGC) was calculated as the difference between the glucose concentrations before and after the cultivation performance. The cell density (CD) of the microbial solution was assessed at the end of each scale-up cultivation performance using a hemocytometer with an optical microscope (BX41, Olympus Instruments, Tokyo, Japan).

The total Cd content in the soil was determined using the acid digestion method, which involved the use of a mixture of HNO_3_, HF, and HClO_4_ (10:5:1, vol/vol). The Cd ion concentration was then measured using an inductively coupled plasma–optical emission spectrometer (ICP–OES, Optima 5300DV, PerkinElmer, Shelton, CT, USA). The diethylenetriamine pentaacetic acid-soluble Cd (DTPA-Cd) content in the soils was evaluated using a soil-to-liquid mass ratio of 1:5 with a diethylenetriamine pentaacetic acid (DTPA) solution mixture. The mixture contained DTPA (0.005 M), triethanolamine (0.1 M), and CaCl_2_ (0.01 M) [24]. The soil removal efficiencies of total Cd and DTPA-Cd were calculated using the formula: removal efficiency (%) = (M_1_ − M_2_)/M_1_ × 100. Here, M_1_ represents the Cd quantity in the soils before the bioremediation treatment, and M_2_ represents the Cd quantity in the soils after the bioremediation treatment.

### 2.5. Microbial Community Analysis

The microbes from microbial solutions (50 mL) in each scale-up cultivation stage were centrifuged at 12,000× *g* for 15 min. Total genomic DNA was extracted using the E.Z.N.A. Water DNA kit (Omega BioTek Inc., Norcross, GA, USA). The quality and quantity of the extracted DNA were assessed using a NanoDrop 2000 Spectrophotometer (Bio-Rad Laboratories Inc., Hercules, CA, USA). For bacterial analysis, the V4~V5 region of the bacterial 16S rRNA gene was amplified using the 515F (5′−GTGCCAGCMGCCGCGGTAA−3′) and 907R (5′−CCGTCAATTCCTTTGAGTTT−3′) primers [25]. For fungal analysis, the ITS region of the fungal rRNA gene was amplified using the ITS1F (5′−CTTGGTCATTTAGAGGAAGTAA−3′) and ITS2R (5′−GCTGCGTTCTTCATCGATGC−3′) primers [26]. The PCR reaction systems and amplification procedure followed a previously published protocol [27]. The PCR products from three replicates of the same sample were pooled and purified using the E.Z.N.A.TM Gel Extraction Kit (Omega BioTek Inc., USA). The purified amplicons from all samples were then sent to E-Gene Biotech Co., Ltd. (Shenzhen, China) for PE300 sequencing on the Illumina HiSeq platform.

The raw sequencing data were merged using fast length adjustment of short reads (FLASH) [28] and assigned to each sample based on the specific barcodes. Low-quality reads were filtered and removed using QIIME’s quality filters to eliminate chimeras [29]. The remaining high-quality sequences were binned into operational taxonomic units (OTUs) at a 97% similarity level using Uparse [30]. The representative sequence for each OTU was selected as the most frequent sequence. Taxonomic information for bacteria and fungi was annotated using the Ribosomal Database Project’s classifier [31] against the SILVA 132 database [32], with a confidence threshold of 0.8~1.0. The assigned sequences for bacteria ranged from 23,731 to 53,194, and for fungi, from 30,372 to 59,959. To normalize the read counts in the OTU table across all samples, the minimum sequence numbers were selected.

### 2.6. Statistical Analysis

One-way ANOVA was conducted to analyze the significance of differences between means, with a significance level of *p* < 0.05, following the least significant difference test (LSD). Error bars accompanied by lowercase letters were employed to indicate statistically significant variations in mean values across distinct groups. Bacterial and fungal alpha-diversities were evaluated using the abundance-based coverage estimator (ACE) and Shannon indices. The microbial beta-diversity was assessed using principal coordinate analysis (PCoA) based on the Bray-Curtis distance metrics. To estimate the contributions of the bioreactor parameter and solution property to the microbial community, variation partitioning analysis (VPA) was performed. Pearson correlation and the Spearman test were used to determine correlations between microbial community data, the bioreactor parameter, and the solution property. Partial least square path modeling (PLSPM) was employed to analyze the effects of bioreactor expansion on the microbial community, solution property, and soil Cd removal. These statistical analyses were carried out using R software version 3.5.1, with the packages vegan, amap, shape, and diagram.

## 3. Results

### 3.1. Properties of Microbial Solution in Scale-Up Cultivation Process of Mixotrophic Acidophiles

**Figure 2:**
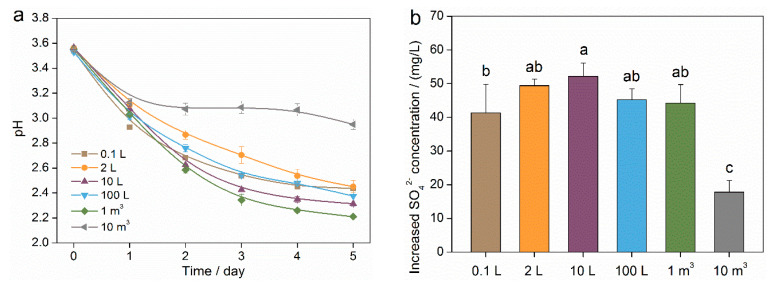

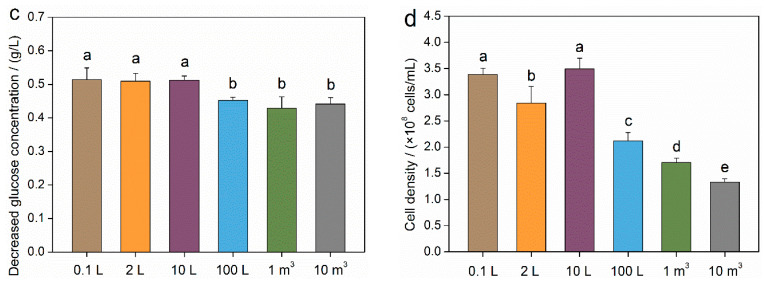
Properties of microbial solutions during the scale-up cultivation process of mixotrophic acidophiles. (**a**) Solution pH. (**b**) Increased sulfate radical concentration. (**c**) Decreased glucose concentration. (**d**) Cell density. Different small letters indicate significant (*p* < 0.05) differences according to the LSD test.

During the scale-up cultivation of mixotrophic acidophiles, the properties of microbial solutions underwent significant changes, especially in the 10 m^3^ stage (Figure 2). When the bioreactor volume increased from 0.1 L to 1 m^3^, the solution pH decreased by 1.1~1.3 units. However, in the 10 m^3^ stage, the solution pH only decreased by 0.6 units within a five-day period (Figure 2a). The bioreactor expansion from the 2 L stage to the 1 m^3^ stage consistently increased the concentration of sulfate radicals (ISC) (Figure 2b). However, in the 10 m^3^ stage, the ISC was significantly lower (*p* < 0.05) compared to the 10 L stage, with a decrease of 65.9% in five days. While no significant (*p* > 0.05) variations in decreased glucose concentration (DGC) were observed for the small system (from 0.1 L stage to 10 L stage) or the large system (from 100 L stage to 10 m^3^ stage), the large system exhibited a significant (*p* < 0.05) decrease in DGC compared to the small system (Figure 2c). The effect of bioreactor expansion on cell density (CD) was consistent between the 0.1 L stage and the 10 L stage (*p* > 0.05) (Figure 2d), but a significant (*p* < 0.05) downward trend in CD was observed with an increase in bioreactor volume.

### 3.2. Microbial Community Diversities and Compositions in Scale-Up Cultivation Process of Mixotrophic Acidophiles

**Figure 3:**
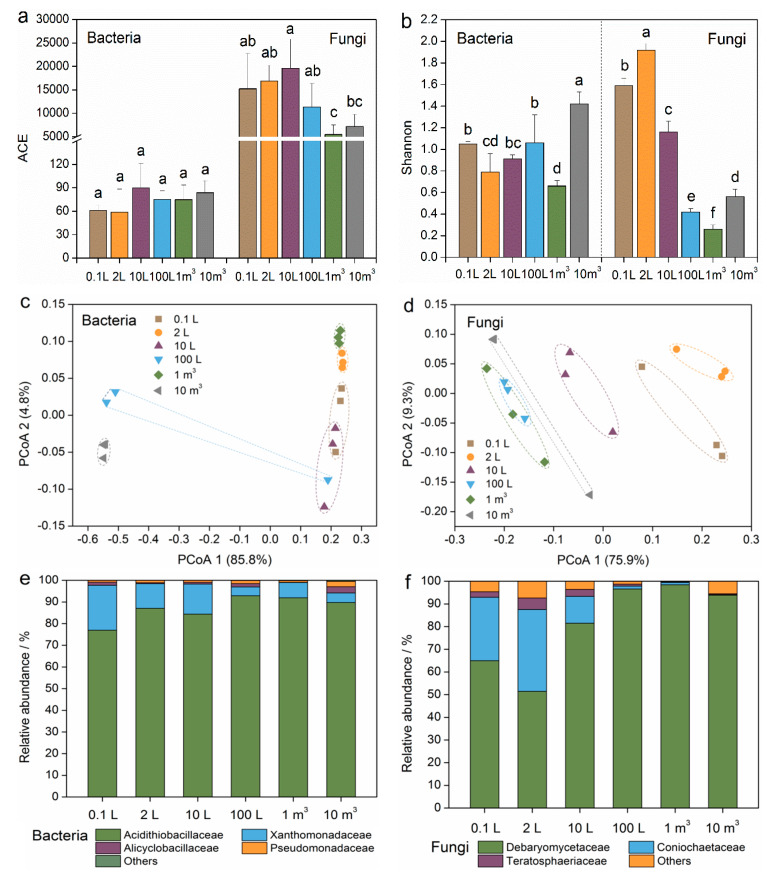
Microbial community diversities and compositions during the scale-up cultivation process of mixotrophic acidophiles. (**a**,**b**) Bacterial and fungal community alpha-diversity, indicated by ACE and Shannon indices. (**c**,**d**) Bacterial and fungal community beta-diversity, indicated by principal coordinate analysis (PCoA) based on the Bray-Curtis distance metrics. (**e**,**f**) Dominant bacteria and fungi at the family level. Different small letters indicate significant (*p* < 0.05) differences according to the LSD test.

The ACE indices did not show significant differences (*p* < 0.05) in the scale-up cultivation process for the bacterial community (Figure 3a). However, the Shannon indices in the 1 m^3^ stage and 10 m^3^ stage were significantly (*p* < 0.05) lower and higher, respectively, than those of the other cultivation stages (Figure 3b). The richness and diversity of microbial OTUs in the fungal community were reduced with bioreactor expansion (Figure 3a,b). The Shannon indices displayed a clear downward trend with the expansion of the cultivation system. Principal coordinate analysis (PCoA) showed inverse changes between the bacterial and fungal communities for the 0.1 L, 2 L, and 10 L stages. The bacterial community structures were not altered in the 0.1 L, 2 L, 10 L, or 1 m^3^ stages based on the PCoA 1 axis (explaining 85.8% of the total variation), but they were different from the bacterial communities in the 10 m^3^ stage (Figure 3c). Bioreactor expansion significantly changed the solution’s fungal community structures for the 0.1 L, 2 L, 10 L, and 10 m^3^ stages (Figure 3d), but it had no effect on the fungal community structures in the 100 L or 1 m^3^ stages (PCoA 1 axis, explaining 75.9% of the total variation).

The relative abundances of dominant fungi at the family level were more sensitive to the expansion of the cultivation system (Figure 3e,f). Within the scale-up cultivation process, the relative abundance of *Acidithiobacillaceae* increased from 77.0% (0.1 L stage) to 89.8% (10 m^3^ stage), while *Xanthomonadaceae* decreased from 20.7% (0.1 L stage) to 4.4% (10 m^3^ stage) (Figure 3e). Additionally, the relative abundances of *Alicyclobacillaceae* and *Pseudomonadaceae* remained stable, ranging from 0.9% to 5.2% in all cultivation systems. For fungal community composition, family-level fungi including *Debaryomycetaceae*, *Coniochaetaceae*, and *Teratosphaeriaceae* were the dominant taxa in the small stages of 0.1 L, 2 L, and 10 L (Figure 3f). However, bioreactor expansion remarkably enriched the family *Debaryomycetaceae*, which accounted for more than 93% of the relative abundance in the large stages of 100 L, 1 m^3^, and 10 m^3^.

### 3.3. Soil Removal Efficiencies of Total Cd and DTPA-Cd Using Mixotrophic Acidophiles in Different Scale-Up Cultivation Process

**Figure 4:**
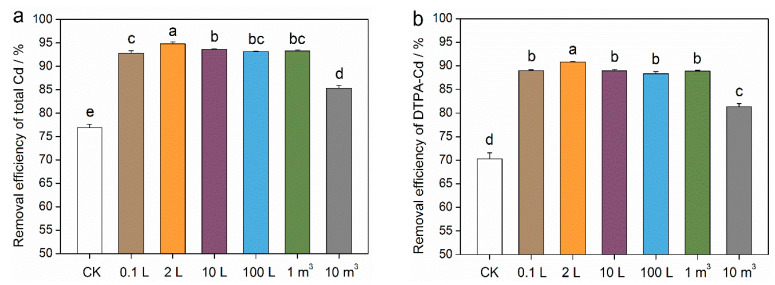
Soil Cd removal efficiencies using microbial solutions of mixotrophic acidophiles during different scale-up cultivation process. (**a**) Total Cd. (**b**) Diethylenetriamine pentaacetic acid-soluble Cd (DTPA-Cd). Different small letters indicate significant (*p* < 0.05) differences according to the LSD test.

The removal efficiencies of total Cd from the 0.1 L group to the 1 m^3^ group exceeded 92.8%, and were significantly (*p* < 0.05) higher than those of the CK group (76.9%) (Figure 4a). The removal efficiency of total Cd in the 10 m^3^ group (85.3%) was also significantly (*p* < 0.05) higher than that in the CK group, but it was the lowest among all the biological groups. The removal efficiency of soil DTPA-Cd followed a similar trend to total Cd extraction, reaching 89.0% (0.1 L group), 90.8% (2 L group), 89.0% (10 L group), 88.3% (100 L group), and 88.9% (1 m^3^ group), respectively (Figure 4b). These values were significantly (*p* < 0.05) higher than those in the CK group (70.2%). The removal efficiency of DTPA-Cd in the 10 m^3^ group was 81.3%, which was also significantly (*p* < 0.05) higher than that in the CK group.

### 3.4. Links between Microbial Solution Properties, Solution Microbes, and Cd Removal Efficiencies

**Figure 5:**
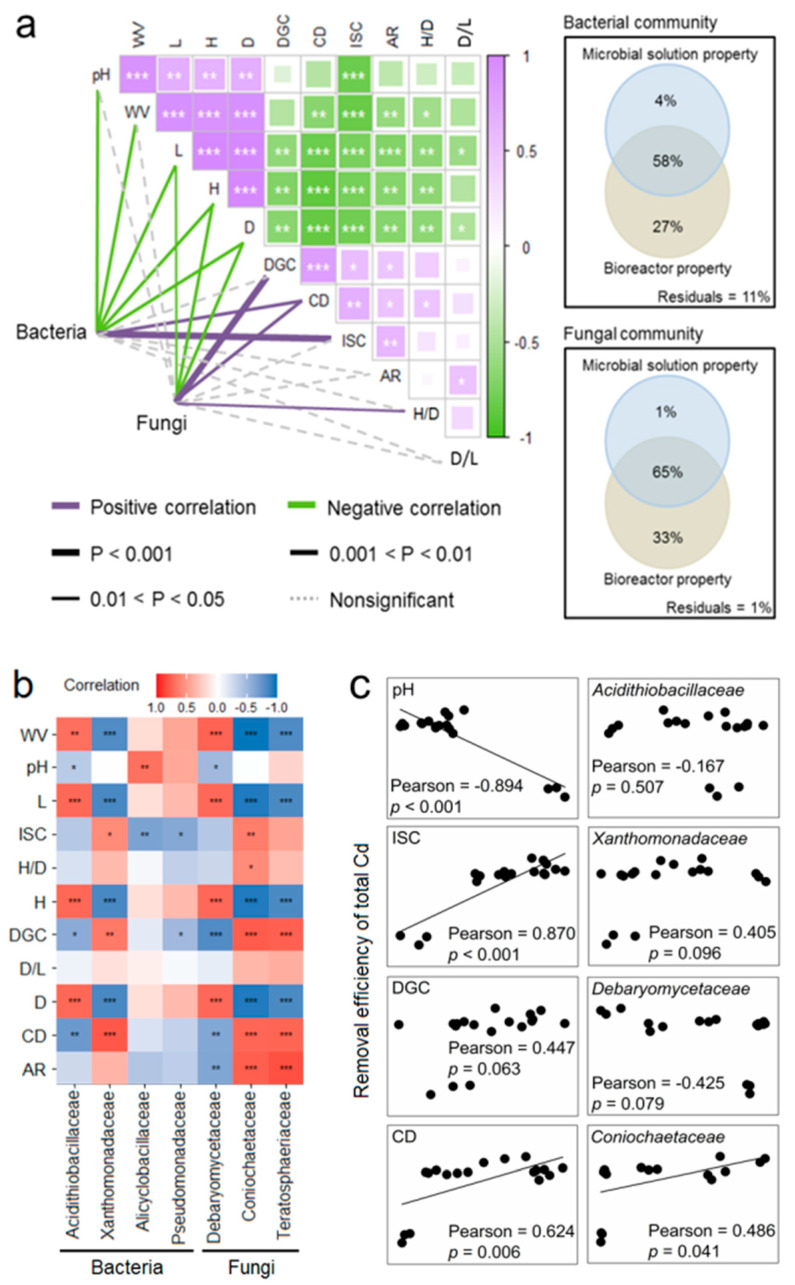
Correlation analysis between microbial community data, bioreactor parameter, and solution property. (**a**) Microbial community structure, bioreactor parameter, and solution property. (**b**) Dominant microbe, bioreactor parameter, and solution property. (**c**) Soil Cd removal, solution property, and dominant microbe. WV, work volume; TV, total volume; H, bioreactor height; D, bioreactor diameter; L, paddle length; AR, agitator agitation rate; ISC, increased sulfate radical concentration; DGC, decreased glucose concentration; CD, cell density. Significances of these correlations are indicated by *** when *p* < 0.001, ** when *p* < 0.01, and * when *p* < 0.05.

The results of the Spearman test revealed that the solution pH had a significant (*p* < 0.01) positive correlation with bioreactor expansion, including the WV, L, H, and D of the bioreactor (Figure 5a). The solution DGC, CD, and ISC all displayed significant (*p* < 0.05) positive associations with the AR of the paddle. Furthermore, ISC showed a negative correlation with the solution pH (*p* < 0.001), but a positive correlation with DGC (*p* < 0.05). Both ISC and DGC were significantly (*p* < 0.01) positively correlated with the solution CD. Variation partitioning analysis (VPA) indicated that the bioreactor parameter and solution property accounted for 27% and 4% of the variation in the bacterial community, respectively, while they accounted for 33% and 1% of the variation in the fungal community (Figure 5a). Specifically, bioreactor expansion (WV, L, H, and D) showed significant (*p* < 0.05) correlations with the bacterial community, as revealed by PCoA 1. Additionally, the bacterial community was significantly (*p* < 0.05) associated with solution pH, CD, and DGC. As for the fungal community, parameters such as the L, H, D, and H/D of the bioreactor, as well as DGC and CD of the solution, displayed stronger relationships with variations in fungal composition.

The WV, L, H, and D of the bioreactor parameters showed significant (*p* < 0.01) correlations with these dominant taxa (Figure 5b). The solution pH showed a significant (*p* < 0.05) negative relationship with the relative abundances of *Acidithiobacillaceae* and *Debaryomycetaceae*, but a significant (*p* < 0.01) positive relationship with *Alicyclobacillaceae*. Solution DGC and CD were significantly (*p* < 0.05) associated with the relative abundance of most dominant family-level microbes. The bioleaching efficiency of total Cd showed significant positive correlations with the solution ISC (*p* < 0.001) and CD (*p* = 0.006), but a significant negative correlation with the solution pH (*p* < 0.001) (Figure 5c). However, dominant taxa such as *Acidithiobacillaceae*, *Xanthomonadaceae*, and *Debaryomycetaceae* did not show significant correlations with the total Cd removal. Only the relative abundance of *Coniochaetaceae* showed a significantly (*p* < 0.05) positive correlation with the total Cd removal.

**Figure 6:**
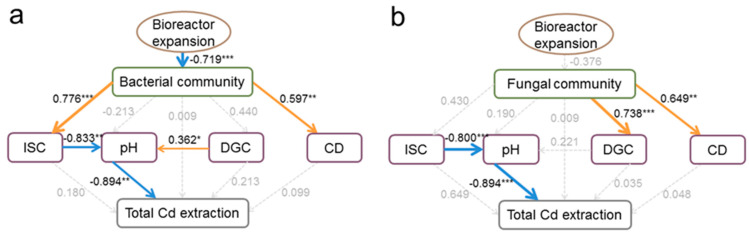
Effects of bioreactor expansion on microbial community and soil Cd extraction, indicated by partial least square path models. (**a**) Bacteria. (**b**) Fungi. Goodness of fit of the modeling was 0.4072. Red and blue arrows represent the significant positive and negative path coefficients. Gray dashed indicate the correlations at a non-significant level. ISC, increased sulfate radical concentration; DGC, decreased glucose concentration; CD, cell density. The significances of these correlations are indicated by *** when *p* < 0.001, ** when *p* < 0.01, and * when *p* < 0.05.

The results of partial least square path modeling (PLSPM) showed that the increase in bioreactor volume had a significant (*p* < 0.001) impact on the bacterial community structure with the scale-up cultivation of mixotrophic acidophiles (Figure 6). Additionally, there were significant (*p* < 0.01) links between the solution ISC and CD (Figure 6a). The solution ISC and DGC had significant (*p* < 0.05) direct correlations with the solution pH, which in turn significantly (*p* < 0.01) affected the removal efficiency of the total soil Cd. The differences in fungal community structure were significantly (*p* < 0.01) correlated with the solution DGC and CD (Figure 6b). The solution ISC directly affected the solution pH (*p* < 0.001) and indirectly increased the efficiency of total soil Cd extraction (*p* < 0.001).

**Figure 7:**
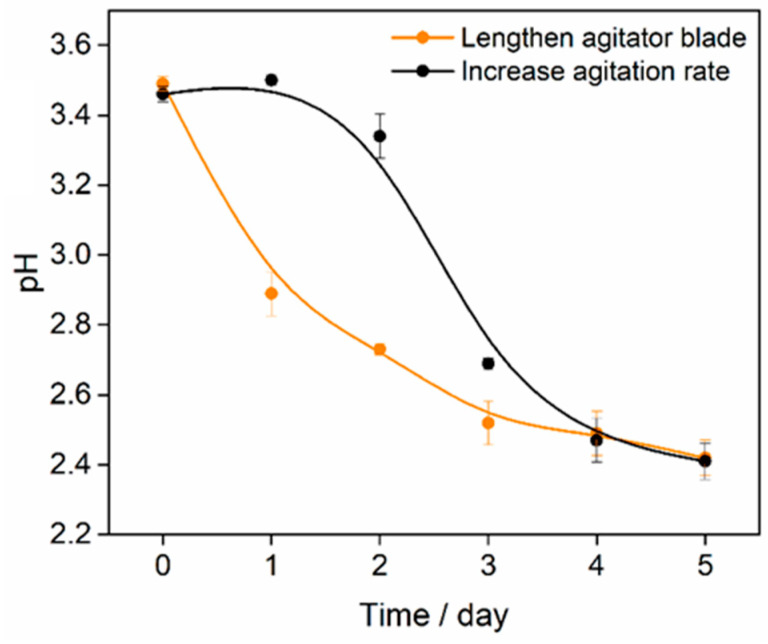
Solution pH changes in the cultivation system of 10 m^3^ after modifying the bioreactor parameters. Red and black lines represent the modifications of lengthening the agitator blade and increasing the agitation rate.

According to Figure 5c and Figure 6, the solution pH was found to play a more significant role in total soil Cd removal. However, the highest solution pH value was obtained in the 10 m^3^ cultivation stage (Figure 2a). To address this issue, modifications were made to the bioreactor parameter for the 10 m^3^ volume by lengthening the agitator blade from 1.2 m to 1.8 m and increasing the agitation rate from 80 rpm to 150 rpm. Mixotrophic acidophiles were then cultured again in the modified bioreactors with a volume of 10 m^3^. The results showed that both modifications, i.e., lengthening the agitator blade and increasing the agitation rate, led to decreases in the solution pH values to 2.4 within five days (Figure 7). However, the modification involving lengthening the agitator blade resulted in a faster reduction rate of solution pH compared to the modification involving increasing the agitation rate.

## 4. Discussion

This study systematically examined the effects of bioreactor expansion on the microbial community succession in mixotrophic acidophiles and the associated changes in solution properties. Additionally, we explored the roles of these factors in Cd removal. The results indicated that bioreactor expansion led to a decrease in microbial growth and biomass accumulation in mixotrophic acidophiles (Figure 2d), hindering the utilization of energy substrates (Figure 2b,c) [33]. Autotrophic microbes were capable of oxidizing elemental sulfur to sulfuric acid and inorganic sulfur compounds [34], while heterotrophic microbes could convert glucose and yeast extract into organic acids and organic substances [35]. Interestingly, we observed a substantial rise in solution pH accompanied by a decrease in solution ISC during the scale-up cultivation process (Figure 5a), suggesting that the decline in solution pH was primarily attributed to the production of sulfuric acid through the oxidation of elemental sulfur.

The alpha-diversity of the fungal community decreased with increasing cultivation volume (Figure 3a,b), suggesting a decline in the variations in fungal species. The similarity in the fungal community structure observed in a large cultivation system (Figure 3d) was potentially due to the simplification of fungal composition (Figure 3f). However, the relationships between the solution property and dominant microbe were particularly noteworthy. The relative abundances of *Acidithiobacillaceae* and *Debaryomycetaceae* showed negative correlations with the solution pH, indicating that their growth and metabolism of these microbes could lead to a reduction in solution pH [36,37]. Given the greater impact of sulfuric acid production on pH variation (Figure 5a), *Acidithiobacillaceae* played a more significant role in reducing solution acidity [38].

The scale-up cultivation process also resulted in changes in the bacterial community, particularly in the 10 m^3^ cultivation stage (Figure 3c). The observed disparity can be traced back to the solubility properties of organic (elemental sulfur) and inorganic (glucose and yeast extract) energy sources, as well as the distinct growth characteristics of bacteria and fungi. Bacterial growth, particularly autotrophic strains, exhibits lower oxygen demand compared to fungal growth [39,40]. For instance, *Acidithiobacillus ferrooxidans* and *Acidithiobacillus thiooxidans*, members of the *Acidithiobacillaceae* family, utilize carbon dioxide as a carbon source [41]. In the bioreactor, the agitation mechanism facilitates bacterial growth by suspending insoluble powdered sulfur, enabling its utilization. However, in the larger 10 m^3^ system, the sulfur powder tended to settle, limiting its accessibility to sulfur-oxidizing bacteria, thus raising the solution pH. On the contrary, organic energy sources dissolve easily, fueling fungal growth, which necessitates substantial oxygen. As the bioreactor volume expanded, the dissolved oxygen concentration declined, thereby decelerating fungal biomass accumulation. In industrial production, measures such as reactor aeration and increasing the rotational speed can be taken to ensure fungal growth. Furthermore, the changes in the bacterial community were correlated with the solution ISC, indicating a limitation in the utilization of elemental sulfur by bacteria (Figure 5a). Meanwhile, the fungal community was correlated with the solution DGC, suggesting a restriction on the utilization of glucose by fungi. These correlations further highlight the impact of bioreactor expansion on the utilization of energy sources by bacteria and fungi.

The PLSPM analysis revealed that bacterial community’s indirect effects on soil total Cd removal were more substantial (Figure 6). Sulfur-oxidizing bacteria in mixotrophic acidophiles convert elemental sulfur to sulfuric acid, lowering the solution pH [42], thus enhancing total Cd extraction efficiency. A low pH of the leaching solution facilitates increased Cd mobility and its release from soils into a stable solution phase. This study observed a decline in microbial solution pH from the 0.1 L to 1 m^3^ stages. Solution pH fluctuations influenced the soil pH, altering Cd adsorption sites, adsorption interface stability, and Cd speciation distribution. As the soil pH decreased, the contents of carbonate-bound Cd, reducible Cd, and residual Cd diminished. These Cd forms were more easily converted to available Cd speciation at a low pH [43,44]. Acid-soluble Cd could be released by the dissolution of solid materials with a pH 5 reagent. The reducible Cd was bound to hydrous oxides of Fe, Mn, and Al in soils. Hydroxylamine hydrochloride (E^0^ = −1.87 V and pH 2.0) could extract this fraction. Residual Cd was encapsulated in a crystalline lattice of silicate and primary and secondary minerals, requiring strong acid digestion for release. This study employed low-pH microbial solutions (pH 2.5) for the bioremediation of Cd-contaminated soils. Consequently, Cd compounds were transformed into Cd^2+^ in the solution and then separated from the soils, reducing the total soil Cd and DTPA-Cd levels.

The solution pH exhibited a significant negative correlation with the removal efficiency of total soil Cd (Figure 5c and Figure 6). Therefore, it was necessary to lower the solution pH during the scale-up cultivation process, particularly at the 10 m^3^ stage. The solution pH was positively correlated with the agitator blade’s length. The high solution pH observed in the 10 m^3^ stage was due to the inadequate blade length to suspend sulfur powder effectively [45]. Although the solution pH was not significantly correlated with AR, AR showed a positive correlation with solution DGC and ISC. Consequently, the bioreactor parameters in the 10 m^3^ stage were readjusted by extending the agitator blade and boosting the agitation rate. As a result, the solution pH values were reduced in both modified bioreactors, with the longer blade resulting in a more rapid pH decline. However, considering energy conservation, increasing the agitation rate could further increase the electrical energy and strain stirring machine. Therefore, it was advised to prioritize extending the agitator blade length as the preferred modification strategy for the 10 m^3^ bioreactor.

The study’s results demonstrate potential for practical implementation, as industrial-scale cultivation of functional strains or microflora has been successfully realized. Mixotrophic acidophiles exhibit a promising capacity to meet remediation demands at the field level. A microbial cultivation facility can strategically be situated near contaminated rice paddies, with the microbial solutions disseminated through irrigation channels or carriers. Irrigation water serves as a medium for cultivating these microorganisms. To replace the flask mixing used in this study, conventional tillage methods liking plowing can be employed to blend the microbial solution with contaminated soil, followed by natural sedimentation. It is crucial to avoid deep soil disruption during mixing process, necessitating the development of automated machinery for consistent-depth plowing for solid–liquid mixing. Supernatants can be drained, separated, and collected in a reservoir lined with impermeable membranes to prevent leakage. Incorporating heavy-metal adsorbents into the reservoir aids in capturing Cd^2+^ from the supernatant. The purified liquid can be repurposed for microbial consortium cultivation, facilitating the transition of mixotrophic acidophilic remediation from laboratory to field-scale implementation.

## 5. Conclusions

This study examined the effects of bioreactor expansion on the succession of mixotrophic acidophiles and its implications for soil Cd remediation. Bioreactor expansion resulted in a delay in sulfur and glucose oxidations and reduced a less pronounced decrease in solution pH and cell density. Bioreactor expansion significantly decreased fungal alpha-diversity, altered the community structure, and simplified the fungal community’s composition. Bacterial diversity and community structure displayed more substantial variations between the 10 m^3^ stage and other cultivation scales. The family-level microbes of *Acidithiobacillaceae* and *Debaryomycetaceae* dominated the bacterial and fungal communities throughout the scale-up process. The indirect effects of mixotrophic acidophiles played a significant role in soil Cd removal. Bacterial community shifts with the changes in bioreactor volume decreased the solution pH, indirectly enhancing Cd removal efficiency. To optimize the 10 m^3^ bioreactor for efficient Cd remediation, it was suggested to increase the agitator blade length. This study offers new insights into the relationships between microbial community dynamics and Cd removal, shedding light on the bioremediation of Cd-contaminated soils in agricultural systems.

## Figures and Tables

**Table 1:** Chemical properties of Cd-contaminated soils used in this study (*n* = 3).

Item	Mean Value ± SD
pH	6.23 ± 0.06
Organic matter (g/kg)	17.3 ± 0.5
Total N (g/kg)	1.74 ± 0.12
Total P (g/kg)	0.736 ± 0.056
Total K (g/kg)	12.7 ± 0.3
Cu (mg/kg)	21.2 ± 0.1
Cd (mg/kg)	13.2 ± 0.2
DTPA-Cd (mg/kg)	7.41 ± 0.83
Pb (mg/kg)	27.8 ± 2.2
Cr (mg/kg)	125 ± 22
Mn (mg/kg)	304 ± 11

## Data Availability

The raw data supporting the conclusions of this article will be made available by the authors upon request.

## References

[1] Ren S., Song C., Ye S., Cheng C., Gao C. (2022). The spatiotemporal variation in heavy metals in China’s farmland soil over the past 20 years: A meta-analysis. Sci. Total Environ..

[2] Liu X., Chen S., Yan X., Liang T., Yang X., El-Naggar A., Liu J., Chen H. (2021). Evaluation of potential ecological risks in potential toxic elements contaminated agricultural soils: Correlations between soil contamination and polymetallic mining activity. J. Environ. Manag..

[3] Bind A., Kushwaha A., Devi G., Goswami S., Sen B., Prakash V. (2019). Biosorption valorization of floating and submerged macrophytes for heavy-metal removal in a multi-component system. Appl. Water Sci..

[4] Shahid M., Dumat C., Khalid S., Niazi N., Antunes P. (2017). Cadmium bioavailability, uptake, toxicity and detoxification in soil-plant system. Rev. Environ. Contam. Toxicol..

[5] Dutta M., Kushwaha A., Kalita S., Devi G., Bhuyan M. (2019). Assessment of bioaccumulation and detoxification of cadmium in soil-plant-insect food chain. Bioresour. Technol. Rep..

[6] Li Y., Sun J., Qian J., Huang T., Su F. (2023). Study on the remediation of cadmium/mercury contaminated soil by leaching: Effectiveness, conditions, and ecological risks. Water Air Soil Pollut..

[7] Fonti V., Dell’Anno A., Beolchini F. (2016). Does bioleaching represent a biotechnological strategy for remediation of contaminated sediments?. Sci. Total Environ..

[8] Wang L., Zhang Q.Y., Liao X.Y., Li X.H., Zheng S.N., Zhao F.H. (2021). Phytoexclusion of heavy metals using low heavy metal accumulating cultivars: A green technology. J. Hazard. Mater..

[9] Verma S., Bhatt P., Verma A., Mudila H., Prasher P., Rene E. (2023). Microbial technologies for heavy metal remediation: Effect of process conditions and current practices. Clean Technol. Environ. Policy.

[10] Srichandan H., Mohapatra R.K., Parhi P.K., Mishra S. (2019). Bioleaching approach for extraction of metal values from secondary solid wastes: A critical review. Hydrometallurgy.

[11] Potysz A., Van Hullebusch E.D., Kierczak J. (2018). Perspectives regarding the use of metallurgical slags as secondary metal resources—A review of bioleaching approaches. J. Environ. Manag..

[12] Raffa C., Chiampo F., Shanthakumar S. (2021). Remediation of metal/metalloid-polluted soils: A short review. Appl. Sci..

[13] Bosecker K. (1997). Bioleaching: Metal solubilization by microorganisms. FEMS Microbiol. Rev..

[14] Brandl H. (2001). Microbial leaching of metals. Biotechnology.

[15] Acharya C., Kar R., Sukla L. (1998). Short Communication: Leaching of chromite overburden with various native bacterial strains. World J. Microbiol. Biotechnol..

[16] Zhu J., Zhang J., Li Q., Han T., Hu Y., Liu X., Qin W., Chai L., Qiu G. (2014). Bioleaching of heavy metals from contaminated alkaline sediment by auto- and heterotrophic bacteria in stirred tank reactor. Trans. Nonferrous Met. Soc. China.

[17] Hao X., Zhu P., Zhang H., Liang Y., Yin H., Liu X., Bai L., Liu H., Jiang H. (2019). Mixotrophic acidophiles increase cadmium soluble fraction and phytoextraction efficiency from cadmium contaminated soils. Sci. Total Environ..

[18] González-Toledo S., Domínguez-Domínguez J., García-Almendárez B., Prado-Barragán L., Regalado-González C. (2010). Optimization of nisin production by *Lactococcus lactis* UQ2 using supplemented whey as alternative culture medium. J. Food Sci..

[19] Brennan L., Owende P. (2010). Biofuels from microalgae-A review of technologies for production, processing, and extractions of biofuels and co-products. Renew. Sustain. Energy Rev..

[20] Ren J., Lin W., Shen Y., Wang J., Luo X., Xie M. (2008). Optimization of fermentation media for nitrite oxidizing bacteria using sequential statistical design. Bioresour. Technol..

[21] Modiri S., Kermanshahi R., Soudi M., Dad N., Ebadi M., Zahiri H., Noghabi K. (2021). Growth optimization of *Lactobacillus acidophilus* for production of antimicrobial peptide acidocin 4356: Scale up from flask to lab-scale fermenter. Iran. J. Biotechnol..

[22] Ren H., Zentek J., Vahjen W. (2012). Optimization of production parameters for probiotic *Lactobacillus* strains as feed additive. Molecules.

[23] Zhang B., Zhang Y., Teng Y., Fan M. (2015). Sulfate radical and its application in decontamination technologies. Crit. Rev. Environ. Sci. Technol..

[24] Yuan B., Huang L., Liu X., Bai L., Liu H., Jiang H., Zhu P., Xiao Y., Geng J., Liu Q. (2022). Application of mixotrophic acidophiles for the bioremediation of cadmium-contaminated soils elevates cadmium removal, soil nutrient availability, and rice growth. Ecotoxicol. Environ. Saf..

[25] Harris J., Kelley S., Pace N. (2004). New perspective on uncultured bacterial phylogenetic division OP11. Appl. Environ. Microbiol..

[26] Cheung M., Au C., Chu K., Kwan H., Wong C. (2010). Composition and genetic diversity of picoeukaryotes in subtropical coastal waters as revealed by 454 pyrosequencing. ISME J..

[27] Hao X., Bai L., Liu X., Zhu P., Liu H., Xiao Y., Geng J., Liu Q., Huang L., Jiang H. (2021). Cadmium speciation distribution responses to soil properties and soil microbes of plow layer and plow pan soils in cadmium-contaminated paddy fields. Front. Microbiol..

[28] Magoč T., Salzberg S.L. (2011). FLASH: Fast length adjustment of short reads to improve genome assemblies. Bioinformatics.

[29] Caporaso J.G., Kuczynski J., Stombaugh J., Bittinger K., Bushman F.D., Costello E.K., Fierer N., Peña A.G., Goodrich J.K., Gordon J.I. (2010). QIIME allows analysis of high-throughput community sequencing data. Nat. Methods.

[30] Edgar R.C. (2013). UPARSE: Highly accurate OTU sequences from microbial amplicon reads. Nat. Methods.

[31] Wang Q. (2007). Naive Bayesian classifier for rapid assignment of rRNA sequences into the new bacterial taxonomy. Appl. Environ. Microbiol..

[32] Quast C., Pruesse E., Yilmaz P., Gerken J., Glckner F.O. (2012). The SILVA ribosomal RNA gene database project: Improved data processing and web-based tools. Nucleic Acids Res..

[33] Manzoor A., Qazi J., Haq I., Mukhtar H., Rasool A. (2017). Significantly enhanced biomass production of a novel bio-therapeutic strain *Lactobacillus plantarum* (AS-14) by developing low cost media cultivation strategy. J. Biol. Eng..

[34] Nguyen T.H., Won S., Ha M., Duc Nguyen D., Kang H.Y. (2021). Bioleaching for environmental remediation of toxic metals and metalloids: A review on soils, sediments, and mine tailings. Chemosphere.

[35] Deng X., Yang Z., Chen R. (2019). Study of characteristics on metabolism of *Penicillium chrysogenum* F1 during bioleaching of heavy metals from contaminated soil. Can. J. Microbiol..

[36] Vera M., Schippers A., Sand W. (2013). Progress in bioleaching: Fundamentals and mechanisms of bacterial metal sulfide oxidation-part A. Appl. Microbiol. Biotechnol..

[37] Brejová B., Lichancová H., Brázdovič F., Cillingová A., Neboháčová M., Tomáška Ľ., Vinař T., Nosek J. (2017). Draft genome sequence of an obligate psychrophilic yeast, *Candida psychrophila* NRRL Y-17665T. Genome Announc..

[38] Ko M., Park H., Kim K., Lee J. (2013). The role of Acidithiobacillus ferrooxidans and Acidithiobacillus thiooxidans in arsenic bioleaching from soil. Environ. Geochem. Health.

[39] Guezennec A., Joulian C., Jacob J., Bodenan F., D’Hugues P., Hedrich S. (2017). Influence of CO_2_ supplementation on the bioleaching of a copper concentrate from Kupferschiefer Ore. Solid State Phenom..

[40] Witne J.Y., Phillips C.V. (2001). Bioleaching of Ok Tedi copper concentrate in oxygen- and carbon dioxide-enriched air. Miner. Eng..

[41] Jerez C. (2017). Bioleaching and biomining for the industrial recovery of metals. Compr. Biotechnol..

[42] Peng T., Zhou D., Liu Y., Yu R., Qiu G., Zeng W. (2019). Effects of pH value on the expression of key iron/sulfur oxidation genes during bioleaching of chalcopyrite on thermophilic condition. Ann. Microbiol..

[43] Yang S., Li Y., Si S., Liu G., Yun H., Tu C., Li L., Luo Y. (2022). Feasibility of a combined solubilization and eluent drainage system to remove Cd and Cu from agricultural soil. Sci. Total Environ..

[44] Liu C., Lin Y. (2013). Reclamation of copper-contaminated soil using EDTA or citric acid coupled with dissolved organic matter solution extracted from distillery sludge. Environ. Pollut..

[45] Guo Z., Zhang L., Cheng Y., Xiao X., Pan F., Jiang K. (2010). Effects of pH, pulp density and particle size on solubilization of metals from a Pb/Zn smelting slag using indigenous moderate thermophilic bacteria. Hydrometallurgy.

